# Real-Time PCR Detection of Candida Species in Biopsy Samples from Non-Smokers with Oral Dysplasia and Oral Squamous Cell Cancer: A Retrospective Archive Study

**DOI:** 10.3390/cancers15215251

**Published:** 2023-11-01

**Authors:** Betül İlhan, Caner Vural, Ceyda Gürhan, Cansu Vural, Ali Veral, Petra Wilder-Smith, Güven Özdemir, Pelin Güneri

**Affiliations:** 1 Department of Oral & Maxillofacial Radiology, Faculty of Dentistry, Ege University, 35040 İzmir, Türkiye; ilhanbetul@yahoo.com (B.İ.); pelin.guneri@ege.edu.tr (P.G.); 2 Molecular Biology Section, Department of Biology, Faculty of Science, Pamukkale University, 20160 Denizli, Türkiye; canervural@gmail.com; 3 Department of Oral & Maxillofacial Radiology, Faculty of Dentistry, Muğla Sıtkı Koçman University, 48000 Muğla, Türkiye; cydgrhn@gmail.com; 4 Basic and Industrial Microbiology Section, Department of Biology, Ege University, 35040 İzmir, Türkiye; caansudogan@gmail.com (C.V.); ozdemirguven@gmail.com (G.Ö.); 5 Department of Medical Pathology, Faculty of Medicine, Ege University, 35040 İzmir, Türkiye; ali.veral@ege.edu.tr; 6 Beckman Laser Institute, University of California Irvine, Irvine, CA 92697, USA

**Keywords:** oral cancer, *Candida albicans*, *Candida tropicalis*, non-albicans species, tissue biopsy, Real-Time PCR

## Abstract

**Simple Summary:**

The current investigation utilized Real-Time PCR to detect the presence of five distinct *Candida* sp. in oral biopsy tissue samples obtained from non-smoker patients with dysplasia, carcinoma in situ, OSCC, and histologically benign lesions. Our results demonstrated a significant increase in the levels of *C. albicans* and *C. tropicalis* in the mild/moderate dysplasia group when compared to both the healthy group and the carcinoma in situ and OSCC cohorts. We noted a consistent coexistence of these two microorganisms, suggesting a potential shift from a commensal state to an opportunistic pathogen, particularly in relation to the onset of oral neoplasia.

**Abstract:**

The impact of *Candida* sp. in the development of oral cancer remains uncertain and requires sensitive analytical approaches for clarification. Given the invasive capabilities of these microorganisms in penetrating and invading host tissues through hyphal invasion, this study sought to detect the presence of five *Candida* sp. in oral biopsy tissue samples from non-smoker patients. Samples were obtained from patients at varying stages of oral carcinogenesis, including dysplasia, carcinoma in situ, OSCC, and histologically benign lesions, and analyzed using Real-Time PCR. Oral tissue samples from 80 patients (46 males and 34 females) were included. Significantly higher *C. albicans* presence was detected in the mild/moderate dysplasia group compared to the healthy (*p* = 0.001), carcinoma in situ (*p* = 0.031) and OSCC groups (*p* = 0.000). Similarly, *C. tropicalis* carriage was higher in tissues with mild/moderate dysplasia compared to healthy (*p* = 0.004) and carcinoma in situ (*p* = 0.019). Our results showed a significant increase in the presence of *C. albicans* and *C. tropicalis* within the mild/moderate dysplasia group compared to other cohorts. Coexistence of these two microorganisms was observed, suggesting a potential transition from a commensal state to an opportunistic pathogen, which could be particularly linked to the onset of oral neoplasia.

## 1. Introduction

The oral cavity presents a unique environment in the context of the microbiome, with a diverse range of habitats that support a mixture of microbial communities including bacteria and fungi. With the contribution of substances found in saliva, these microbial populations come together to form biofilms on the surfaces of both oral mucosa and teeth. Despite the presence of various species within these biofilm structures, a state of equilibrium is maintained through complex interactions between these microorganisms and the host. This helps prevent any deviations from this balance, ensure that the microbial communities remain in a harmonious state, and prevent the onset of pathological conditions [1,2]. Bacteria are the predominant members of the oral microbiome with approximately 1000 bacterial strains being cultivated to date [3,4]. Oral fungal species make up a small percentage of this population, and the most commonly isolated species is *C. albicans* followed by *Cladosporium*, *Aureobasidium*, *Saccharomycetales*, *Aspergillus*, and *Fusarium* [2,5]. The oral microbiome is unique among individuals and reflects diversities such as age, habits, nutrition, salivary pH, the smoking status of the host, and geographical locations [6]. In health, multiplication of the organism is normally kept under control by specific and non-specific defense mechanisms of the saliva and oral mucosa as well as by competition among oral endogenous microflora [6,7]. Several studies suggest that the risk of developing various diseases including oral cancer may increase and the malignant transformation may be initiated when this balance is disturbed (dysbiosis) [3,6,7,8,9,10,11,12]. Indeed, microbial infections have been considered among the contributing—if not one of the major—causes of chronic inflammation, which leads to carcinogenesis via the dysregulation of cellular growth observed in the cellular micro-environment as increased cell proliferation, decreased apoptosis, mutagenesis, oncogene activation, and angiogenesis [13]. Chronic oral infectious conditions that result in chronic inflammation, especially chronic periodontitis [11,14] and poor oral hygiene [15], have been related to the development of oncological diseases. Recent research to identify the bacterial signatures in the oral microbiome of patients with oral squamous cell carcinoma (OSCC) implicated over-representation of, in particular, *Fusobacterium*, *Porphyromonas*, *Capnocytophaga*, *Prevotella*, and *Peptostreptococcus* species [11,16,17,18,19,20,21,22], yet the determination of a conclusive causal relationship attributed to the oral microbiome has sparked controversy [23], probably due to the inherent disparities present in anatomical sites and methodologies of microbial sampling, and the diverse array of evaluative techniques employed [24].

Oral *Candida* infection has also been associated with the development of OSCC [11,21,23,25,26]. In early studies, Cawson [25] suggested that *Candida* infection itself is a cause of leukoplakia, mainly due to the invasion of epithelium by *Candida* organisms and resulting hyperplasia, and Williamson [26] reported two cases of oral squamous cell carcinoma (OSCC) with mycological findings consistent with chronic hyperplastic candidiasis. These pioneering reports were further supported by several epidemiological and descriptive studies that revealed significantly higher oral *Candida* carriage among patients with oral epithelial dysplasia (OED) and OSCC compared to healthy subjects [27,28,29,30,31]. The suggested mechanisms for fungal involvement in oral carcinogenesis include the secretion of potential carcinogens, conversion of pro-carcinogens such as acetaldehyde or nitrosamines, destruction of epithelial barriers by secreted proteolytic enzymes, and excessive chronic inflammation [21,32,33,34]. Recent in vitro experimental studies reported that OSCC cells secreted significantly more pro-inflammatory cytokine IL-1β when treated with zymosan (ZYM), a component from the cell wall of *C. albicans* [35], and the progress of OSCC was promoted by the presence of living *C. albicans* via stimulating the production of matrix metalloproteinases (MMPs), tumor metabolites, and tumor-promoting signal pathways [36]. Although these results suggest that *C. albicans* may participate in the complicated process of oral carcinogenesis, chronic inflammation caused by microbial infection is one of the risk factors for tumor development [37]. This hypothesis posits that continuous stimulation of epithelial cells could render them susceptible to malignant transformation, and such persisting cells may be attributed to compromised macrophage and natural killer cell activity induced by the fungal presence [21]. Thus, the severity of inflammatory changes in cancer areas and the virulence of *Candida* isolated from these sites should also be considered in the process of malignant transformation. Contrarily, it has also been reported that the presence of *Candida* in OED and OSCC does not solely justify its role in carcinogenesis, since the increased colonization and prevalence of oral yeast in oral malignant lesions may be entirely coincidental, reflecting an altered local environment [38,39,40,41]. Additionally, if *Candida* sp. were likely to promote carcinogenesis of the oral mucosa, it might be expected that many more patients with chronic mucocutaneous candidiasis would have developed OSCC [30]. Recently, chronic candidiasis was excluded from the list of oral potentially premalignant lesions (OPMDs) in the fifth chapter of the upcoming fifth edition of World Health Organization Classification of Tumours of the Head and Neck, 2022, due to a lack of supportive evidence regarding its role in the promotion of oral carcinogenesis [42]. Furthermore, as an addition to the discussion of the impact of the microbiome in carcinogenesis, it was suggested that non-albicans *Candida* sp. may also be involved [32], and the identification of these microorganisms from the oral cavity of patients with dysplastic or malignant lesions drew attention as an important determinant of the disease rather than a contaminant only [43,44]. Throughout the pathological process, in the same way as *C. albicans*, non-albicans yeasts also produce carcinogenic amounts of acetaldehyde from ethanol, and *C. glabrata* can perform this by fermentation from glucose. Therefore, colonization by *C. glabrata* and *C. kruseii*, which are resistant to antifungal therapy, requires investigation to assess whether these species may be contributing factors to oral cancer development, especially in patients with no alcohol consumption [45].

Several diagnostic techniques are used to detect *Candida* sp. In the oral environment, the most common ones are microbiological methods that allow the growth of the yeasts on culture media, and histological staining of tissue sections [46,47,48,49]. Each method has both advantages and disadvantages, and the choice of sampling technique is primarily decided by the nature of the lesion [46,47]. When an accessible and defined mucosal lesion is evident, a direct sampling approach should be preferred, whereas in cases with no obvious lesions, an indirect sampling based on saliva specimens or an oral rinse is more acceptable [48]. However, blastospore forms of *Candida* sp. (round or ovoid) are usually found on the surface while the hyphal forms are vertically penetrating the epithelium [48]. Moreover, the presence of *C. albicans* further amplifies and stabilizes effective establishment of *C. glabrata* colonization during co-infection, with the hyphal wall adhesins Als 1 and 3 (agglutinin-like sequence proteins), Hwp1p (hyphal wall protein), and eap1 (enhanced adherence to polystyrene) playing a crucial role in facilitating in vitro adhesion of *C. glabrata* and establishing oro-pharyngeal candidiasis [49]. Considering the ability of these microorganisms to penetrate and invade host tissues through hyphal invasion [2], the role of *Candida* sp. in oral cancer development has yet to be established with sensitive analyses. Therefore, this study aimed to determine the presence of *C. albicans*, *C. kruseii, C. parapsilosis*, *C. tropicalis*, and *C. glabrata* in oral biopsy tissue samples of non-smoker patients with different stages of oral carcinogenesis, namely dysplasia, carcinoma in situ, OSCC, and histologically benign lesions by using Real-Time PCR.

## 2. Materials and Methods

### 2.1. Oral Biopsy and Sample Collection

The study protocol was approved by the Research Ethics Committee, Faculty of Medicine, Ege University (No: 17-7.2/5), and all procedures were performed in compliance with the Helsinki Declaration protocols. Oral biopsy samples from patients who applied to the outpatient clinic of the Faculty of Dentistry, Ege University between January 2021 and December 2021 with suspicious oral mucosal lesions requiring histopathological examination were included in the present study. The inclusion criteria were systemically healthy patients over 18 years old with no smoking habit, no prior history of oral or any other forms of cancer, no clinically evident oral *Candida* infection or recent antifungal therapy, and the presence of an oral mucosal lesion that required a biopsy due to suspicion of dysplasia or cancer. The demographic data and use of dentures among the patients were recorded. After written informed consent was obtained, a biopsy was performed and tissue specimens were formalin-fixed, paraffin-embedded, and stained with hematoxylin and eosin for histological analysis by an oral pathologist with an experience of 30 years. The histological diagnoses were made according to uniform criteria [46]. After histopathological examination and final diagnosis, paraffin-embedded tissue samples were accessed from the archives of the Department of Pathology, Faculty of Medicine, Ege University. A total of 106 samples were identified, 26 of which were considered as not suitable for further analysis due to some degree of specimen damage during the histopathological preparation. A total of 80 tissue samples were included in the study and were classified as benign (no dysplasia), mild/moderate dysplasia (due to the low number of dysplasia cases, mild and moderate dysplasia cases were grouped), or carcinoma in situ and OSSC [46]. The paraffin-embedded tissue samples were cut out and weighed to obtain an average of 50 mg of tissue sample from each patient and used for further analyses.

### 2.2. Growth of Candida Species and Fungal DNA Extraction

*C. albicans*, *C. kruseii, C. parapsilosis*, *C. tropicalis*, and *C. glabrata* were kindly obtained from the Department of Basic and Industrial Microbiology, Faculty of Science, and Department of Infectious Diseases and Clinical Microbiology, Faculty of Medicine, Ege University. *Candida* species were individually inoculated on Sabouraud Dextrose Agar (SDA) using the streak plate method and incubated at 37 °C for 24–48 h. After incubation, colonies were checked for purity and used for further analyses. In order to calculate the amount of *Candida* l DNA, the genomic DNA of each *Candida* sp. were obtained, and a preliminary PCR step was applied. For this, single colonies of each *Candida* sp. were subjected to DNA extraction with a fungal/bacterial DNA isolation kit (Zymo Research, Irvine, CA, USA) according to the user’s manual. Eluted total DNA samples were checked for purity and amount by measuring the A260/280 ratio with a micro-volume spectrophotometer (Nanodrop 2000c, Thermo Scientific, Waltham, MA, USA). DNA extractions were carried out using the FastStart Essential DNA Green Master Kit (Roche Diagnostics GmbH, Penzberg, Germany). Thereafter, initial in vitro DNA amplifications were performed by Real-Time PCR (LightCycler 96, Roche Diagnostics GmbH, Germany) using species-specific primer sets (Table 1). The Real-Time PCR protocol for all extracted DNAs is presented in Table 2.

### 2.3. Total DNA Extraction from Tissue Samples

A Roche High Pure PCR Template Preparation Kit (Roche Diagnostics GmbH, Germany) was used to extract total DNA from tissue samples. After deparaffinization, samples were embedded in graded ethanol solutions of 100%, 80%, 60%, and 40%, respectively, for 10 s for rehydration. Subsequently, deparaffined tissue samples were rehydrated with double-distilled water for 10 s. Each sample was placed into sterile 1.5 μL microcentrifuge tubes and DNA extraction was performed. Briefly, 200 μL Tissue Lysis Buffer and 40 μL reconstituted Proteinase K were added to samples, respectively, and incubated overnight at 37 °C after mixing well. Following overnight incubation, 20 μL reconstituted Proteinase K was added and incubated for 1–2 h at 55 °C. Then, 200 μL Binding Buffer was added to samples, mixed thoroughly, and incubated for 10 min at 70 °C. Thereafter, 100 μL isopropanol was added to each tube and mixed. Washing and elution protocol was performed according to the manufacturer’s instructions. The purity and amount of extracted DNA samples were measured using the A260/280 ratio and a micro-volume spectrophotometer (Nanodrop 2000c, Thermo Scientific, USA).

### 2.4. Quantitative PCR Analysis

A Real-Time PCR (LightCycler 96, Roche Diagnostics GmbH, Germany) device was used to detect 5 *Candida* species in tissue samples and calculate the numbers of these species. In order to perform numerical calculations, PCR was performed with the planned primer sets, using the previously obtained genomic DNAs of each *Candida* sp. as templates (Table 1). Synthetic oligonucleotides obtained through the initial PCR were cleaned using a PCR product clean-up kit (NucleoSpin^®^ Gel and PCR Clean-up Kit, Macherey-Nagel GmbH & Co. KG, Düren, Germany). After the amounts and purity measurements of the DNAs obtained after cleaning were measured with a nanodrop, the molecular amounts of the cleaned DNAs were calculated, and a serial dilution of each DNA sample was made. Those DNA dilutions were then used to generate standard curves in Real-Time PCR. Standard curves were constructed over a range of 101 to 1010 amplicons of target DNA·µL^−1^. Determination of the cycle threshold point (Ct) occurs during the exponential phase of the PCR cycle. The prepared DNA dilutions were studied in a Real-Time PCR device and a standard curve graph was created. Tm values of PCR products made with the primer set used were calculated. The generated standard curve plots were used to calculate the % yield of the PCR reaction.

### 2.5. Detection of Candida Species in Total Tissue DNA Samples

To identify each *Candida* sp. in the overall tissue samples, the primer sets outlined in Table 1 were employed, along with the standard curves detailed in Table 3 to establish their respective properties. The normality of data distribution was analyzed using the Kolmogorov–Smirnov test, which presented non-normal distribution for all *Candida* sp. Data was analyzed using Chi-square, Kruskal-Wallis, and Mann–Whitney tests. Statistical significance was set at *p* < 0.05.

## 3. Results

This study was conducted on 80 oral tissue samples from 80 patients (46 males and 34 females) who met the specified inclusion criteria. No significant differences were observed between the number of males and females among the four histopathological groups (*p* = 0.447) (Table 4). The mean age of males and females was similar (*p* = 0.174), and the difference between the age distribution within the four groups was statistically insignificant (*p* = 0.101). Benign lesions and the samples with mild-moderate dysplasia were observed similarly in males and females. However, carcinoma in situ and OSCC were more common in males (70% and 60%, respectively). Benign lesions and samples showing mild to moderate dysplasia exhibited a similar distribution between males and females (45–55%, and 55–45%, respectively). However, carcinoma in situ and OSCC were found to be more prevalent among males (70% and 60%, respectively) (Table 5).

Table 5 presents the prevalence of *Candida* sp. according to gender and histopathological diagnosis. *C. albicans* was significantly more common among men compared to women (*p* = 0.033), whereas the number of positive samples for *C. glabrata* was statistically higher among females (*p* = 0.033). Gender did not have any discernible impact on the abundance of other *Candida* sp. (*p* > 0.05).

The number of samples with the presence of different *Candida sp*. in each histopathological group is presented in Table 6. Polyphyly was evident in select specimens, with a plurality of patient samples revealing co-occurrence of distinct species, across the entire spectrum of patient sample set; *C. albicans* was detected in 55% of the samples and was followed by *C. glabrata* (45%), *C. kruseii* (41.25%), *C. tropicalis* (12.5%), and *C. parapsilosis* (8.75%). The comparison of the four histopathological groups regarding the number of positive samples for each *Candida* sp. revealed significant differences between the groups for *C. albicans* (*p* = 0.001) and *C. tropicalis* (*p* = 0.004). Significantly higher *C. albicans* presence was detected in the mild/moderate dysplasia group compared to the healthy (*p* = 0.001), carcinoma in situ (*p* = 0.031), and OSCC groups (*p* = 0.000). Similarly, the number of positive samples for *C. tropicalis* was also significantly higher in tissues with mild/moderate dysplasia when compared to healthy (*p* = 0.004) and carcinoma in situ (*p* = 0.019). Although the number of positive samples for *C. glabrata* and *C. kruseii* was higher than *C. albicans* in the OSCC group, these differences did not reach statistical significance (*p* > 0.05).

## 4. Discussion

Due to the complex and multifaceted nature of carcinogenesis, the role of specific or entire oral microbiomes in oral cancer development and prognosis is still a subject of ongoing research [24]. *C. albicans* is one of the primary oral microorganisms whose influence on the development of oral cancer has been under evaluation for several decades [11,21,23,27,28,29,30,31,32,33,34,35,36,37]. The connection between oral mycobiome and carcinogenesis is underscored by the intricate mechanisms through which *C. albicans* invades keratinocytes and surpasses the innate local defense abilities of oral mucosa. Two main mechanisms have been identified [50,51]. The first mechanism involves the degradation of the epithelial cell surface by the aspartic proteases of the fungus which enables the physical movement of the hyphae into the host cells [51,52]. The second mechanism encompasses epithelial cell endocytosis, which is induced by *C. albicans*’ stimulation of the host cell to produce pseudopods to pull the fungus into the cell [51,52,53]. Nonetheless, it has been reported that factors contributing to fungal adhesion, invasion, and destruction extend beyond fungal morphology and activity, encompassing the type and stage of differentiation of keratinocytes as well as keratinocyte adhesins [53]. This highlights the significance of host-related factors being just as critical as the characteristics of the active microbial agent, potentially leading to variations in the presence and severity of fungal infection based on these fundamental features. Given the impracticality of standardizing these factors, it becomes imperative to interpret the findings of each study in the context of its unique sample group. In alignment with this perspective, the scientific literature indeed presents diverse outcomes, predominantly attributable to methodological variations and inherent complexities. These differences encompass a range of factors, including host-related variables such as demographic characteristics, overall and oral health status, genomic traits, lifestyle choices, as well as the histological type and severity of the malignancy. Additionally, technical distinctions, such as variations in sample collection methods (swabs, stimulated/unstimulated whole saliva, biopsy), the sampling site, storage, and DNA isolation techniques can significantly impact research findings [24,30,51,54]. Furthermore, there are notable discrepancies in the composition of microorganisms between the surface epithelium and deep tissue. Certain microorganisms tend to be more prevalent on the surface, while, as implied for *C. albicans* and some non-albicans sp., others demonstrate a higher abundance within the deeper tissues [24,55,56].

The present study employed Real-Time PCR to determine the presence of *C. albicans, C. kruseii*, *C. parapsilosis*, *C. tropicalis*, and *C. glabrata* within oral biopsy tissue samples of non-smoker patients with dysplasia, carcinoma in situ, OSCC, and histologically benign lesions. Real-Time PCR stands as the leading precise molecular technique for ascertaining the etiology of clinical *Candidal* intrusion within blood, tissues, and cultures, and is highly sensitive for the detection of even low amounts of DNA or RNA [47,57]. After histopathological examination and final diagnosis, total DNA extraction was performed from paraffin-embedded tissue samples. The utilization of any portion of fresh tissue immediately following the initial biopsy was excluded from consideration, as it had the potential to compromise the reliability of the histopathological diagnosis. Furthermore, obtaining a second biopsy from the same lesion was deemed ethically untenable. *C. albicans* and non-albicans sp. can also be identified by germ tube information tests or by growth on CHROMagar medium. However, it is essential to note that each of these methodologies necessitates the cultivation of the organism on solid medium for a minimum of 24 h, and frequently, an extended period of 48 h, before such tests can be performed [57]. Therefore, tissue samples collected and processed for pathological diagnosis have become a unique source of archived and morphologically defined disease-specific biological material. This has been further enhanced by the significant advancement in fungal diagnostics, enabled by the ability to extract and identify fungal DNA from paraffin-embedded tissues, including the extraction of quality *Candidal* DNA as reported [38,47,58].

Utilizing previously established and recommended analytical approaches, our findings revealed significant differences among the dysplasia, carcinoma in situ, OSCC, and benign lesion groups concerning the presence of *C. albicans* and *C. tropicalis*. A markedly elevated level of *C. albicans* presence was identified within the mild/moderate dysplasia group compared to the healthy group, as well as the carcinoma in situ and OSCC cohorts. Similarly, the number of positive samples harboring *C. tropicalis* demonstrated a statistically significant elevation within the mild/moderate dysplasia tissue when compared to the benign and carcinoma in situ groups. Although *C. glabrata* was the second most frequently encountered sp. in the studied sample and was more common than *C. albicans* in the OSCC group, these differences did not reach statistical significance. Several studies have used oral rinse or saliva samples, applying CHROMagar for the identification of *Candida* sp. based on colony color and morphology. These investigations have consistently reported a higher carriage of *C. albicans* among subjects with OSCC compared to healthy subjects [28,29,31,44,59,60]. In contrast, using different methodologies such as PAS staining (38) and CFW fluorescence staining [39], or CHROMagar [61] and some sort of growth medium [62], others did not report a direct causative relationship between *C. albicans* and OSCC or epithelial dysplasia. Furthermore, it was reported that non-albicans sp. predominated *C. albicans* in patients with epithelial dysplasia, oral premalignant lesions, and OSCC, and *C. tropicalis* emerged as the most frequently isolated fungi underscoring the significance of non-albicans sp. as an emerging contributor to oral infections [31,32,44]. The findings of this current study are in agreement with some of the existing reports, indicating that there is no predominated presence of *C. albicans* in tissue samples from OSCC patients compared to healthy subjects [38,39,40,62]. However, in cases of mild to moderate dysplasia, *C. albicans* and *C. tropicalis* exhibited the highest prevalence, a trend that exhibited a gradual decline in higher malignancy groups, including carcinoma in situ and OSCC. This may imply that the commensal condition of both fungi might transform into that of an opportunistic pathogen, particularly associated with the initiation of oral neoplasia [63,64].

Recent epidemiological data underscore a notable mycological transformation, wherein *C. albicans*, which is still the predominant etiological agent, is exhibiting a diminishing proportional representation whereas the predominance of alternative species, notably *C. glabrata*, *C. tropicalis*, and *C. parapsilosis*, is ascending [51]. It has been reported that oxidative stress may act as an initiator for *Candidal* morphological alterations [65]. Given that oxidative stress has been identified as one of the microenvironmental changes observed in oral carcinogenesis [66,67], the involvement of *C. albicans* and *C. tropicalis* in the invasion of deep oral mucosal tissue may be linked to the morphological transitions. Despite its morphological similarities to *C. albicans* and its occurrence among oncology patients [51], the influence of *C. tropicalis* on fungal infiltration of epithelial tissues has not been thoroughly explored [51]. In contrast to de Barros et al. [66], who reported an antagonistic relation between *C. tropicalis* and *C. albicans*, a congruent behavior between *C. tropicalis* and *C. albicans* was observed within our sample. The capability of *C. tropicalis* to engender biofilms and its involvement in the origination and severity of candidiasis have been well documented in the literature [51,60,66,67,68]. *C. tropicalis* is a real hypha-former, as are *C. albicans* and C. dubliniensis, and with its ability to invade the host epithelial layers, it may cause endothelial rupture, survive phagocytic cell attack, and form biofilms, which all contribute to *Candidal* infection [68]. Considering that *C. albicans* has similar characteristics and these two fungi are frequently associated with infectious processes, they probably have adaptations to specific environments which also induce morphogenesis and hyphae formation [68]. Additionally, *C. tropicalis* has greater genetic similarity with *C. albicans*, which may be the result of predominant clonal reproduction phylogenetically [68]. The parallel existence of *C. albicans* and *C. tropicalis* can be elucidated through an appraisal of their shared morphogenetic traits and roles in the process of fungal invasion and infection. This observation holds significance in the context of treatment paradigms, particularly considering that *C. tropicalis* demonstrates heightened resistance to antifungal agents, especially azole antifungals [51,55,67,68]. The concurrent prevalence of both species within oral epithelial tissues might potentially compromise the efficacy of antifungal therapy, possibly resulting in the protracted persistence of fungal infections.

Within the context of various groups related to health, dysplasia, carcinoma in situ, and OSCC, there was a noticeable opposition in the occurrence of *C. albicans* and *C. glabrata*, as well as between *C. albicans* and *C. kruseii*. When one of these species exhibited a higher colonization level, the colonization of the other species tended to decrease. This observation aligns with the results reported by others [66,69], where it was noted that *C. kruseii* and *C. glabrata* have the ability to modify or hinder mechanisms associated with in vitro adhesion and biofilm formation of *C. albicans*. Furthermore, the antagonistic interactions between *C. albicans* and *C. glabrata* in Sabouraud dextrose are influenced by strain type, culture medium, and glucose supplementation [69]. Although the synergistic effects of albicans and non-albicans species have been observed [51], it is important to recognize the inherent differences between in vitro models and real-life conditions when applying these findings to clinical practice [51,70].

The key aspect of the present study is the enrollment of only non-smoker patients with oral benign, dysplastic, and OSCC lesions. Given the substantial influence of smoking status on fungal colonization of the oral mucosa [24,38,54,71], our study deliberately focused on non-smokers to eliminate any potential effects of smoking on *Candidal* colonization. However, this stringent criterion inevitably resulted in a limited participant pool (*n* = 80), particularly within the pathological group (including dysplasia, carcinoma in situ, and OSCC), thus constituting the limitation of our study. One aspect we should acknowledge also as a limitation is the lack of information regarding the periodontal health of the patients, which might have had an impact on the colonization of *Candida* sp. When the close association between *C. albicans* and chronic periodontitis is taken into consideration [72,73], the presence of periodontitis and poor oral health might alter the abundance of *C. albicans* and, due to morphogenetic similarity, of *C. tropicalis* in mild/moderate dysplasia, carcinoma in situ, and OSCC.

Nevertheless, the present study has demonstrated the abundance of *C. albicans* and *C. tropicalis* in tissue samples obtained from non-smokers exhibiting oral benign, dysplastic, and OSCC lesions. Both fungi exhibited the highest prevalence in cases of mild to moderate dysplasia, and then gradually declined in higher malignancy groups, suggesting that these microorganisms were present in the initial stages of cellular alteration in the oral epithelium, and this observation may provide insights into the potential involvement of *C. albicans* and *C. tropicalis* in the initiation of oral neoplasia.

## 5. Conclusions

The current study employed Real-Time PCR to determine the presence of five different *Candida* sp. within oral biopsy tissue samples of non-smoker patients with dysplasia, carcinoma in situ, OSCC, and histologically benign lesions. Our findings revealed a markedly elevated level of *C. albicans* and *C. tropicalis* in the mild/moderate dysplasia group compared to the healthy group, as well as the carcinoma in situ and OSCC cohorts. A congruent existence of these two microorganisms was observed, which may imply that the commensal condition of both fungi might transform into that of an opportunistic pathogen, particularly associated with the initiation of oral neoplasia. Prospective studies involving larger and more diverse cohorts are necessary to gain a more comprehensive understanding of the involvement of *Candida* sp., particularly non-albicans types, in the intricate process of oral carcinogenesis.

## Figures and Tables

**Table 1:** Species-specific primers sets and annealing temperatures used in Real-Time PCR.

Target Yeast		Oligonucleotide Sequence (5′ → 3′)	Length (bp)	Annealing Temperature (°C)
*C. albicans*	Calb F	TTTATCAACTTGTCACACCAGA	273	51
	Calb R	ATCCCGCCTTACCACTACCG		
*C. glabrata*	Cgact1 F	GACGGCGATTATGAGTTAGGAG	102	53
	Cgact1 R	GTAGCATCTGTGCAGGTAGTT		
*C. kruseii*	Trfp4 F	AGGCAGCAGACTTGTACCTT	183	54
	Trfp4 R	TGCCCAGTTTCGAGGTGAGA		
*C. tropicalis*	Trf4 F	TGTTGGTGGTCTTGGTGGGT	108	57
	Trf4 R	ACCCCCAAATTGTCTAATGCAC		
*C. parapsilosis*	Sadh F	ACCCGTTGTGAGAAGTGCCA	124	57
	Sadh R	ACCAAGCCTATGTCCGCAACT		

**Table 2:** Real-Time PCR protocol for *Candida* l DNAs.

C. Species	Preincubation Stage	Amplification Stage (45 Cycles)	Melting Stage	Cooling Stage
*C. albicans*	95 °C for 10 min	95 °C for 10 s	95 °C for 10 s	37 °C for 30 s
		51 °C for 10 s	65 °C for 60 s	
		72 °C for 12 s	97 °C for 1 s	
*C. kruseii*	95 °C for 10 min	95 °C for 10 s	95 °C for 10 s	37 °C for 30 s
		54 °C for 10 s	65 °C for 60 s	
		72 °C for 10 s	97 °C for 1 s	
*C. glabrata*	95 °C for 10 min	95 °C for 10 s	95 °C for 10 s	37 °C for 30 s
		53 °C for 10 s	65 °C for 60 s	
		72 °C for 6 s	97 °C for 1 s	
*C. parapsilosis*	95 °C for 10 min	95 °C for 10 s	95 °C for 10 s	37 °C for 30 s
		57 °C for 10 s	65 °C for 60 s	
		72 °C for 6 s	97 °C for 1 s	
*C. tropicalis*	95 °C for 10 min	95 °C for 10 s	95 °C for 10 s	37 °C for 30 s
		57 °C for 10 s	65 °C for 60 s	
		72 °C for 6 s	97 °C for 1 s	

**Table 3:** PCR efficiency values and standard curves constructed with Real-Time PCR for each primer set.

	Primer	Slope	PCR Efficiency (%)	Linearity (R^2^)	y-Intercept
*C. albicans*	Calb F–Calb R	−3.3350	99	0.98	40.05
*C. glabrata*	Cgact1 F–Cgact1 R	−3.3366	99	0.99	35.56
*C. kruseii*	Trfp4 F–Trfp4 R	−3.3300	100	1.00	33.77
*C. tropicalis*	Trf4 F–Trf4 R	−3.3557	99	1.00	33.24
*C. parapsilosis*	Sadh F–Sadh R	−3.3350	99	1.00	36.05

**Table 4:** Age and gender distribution of the study population.

Histopathological Diagnosis	Gender (*n*)		Age (Mean ± SD)		Total (*n* = 80)	
	Male	Female	Male	Female	Age (Mean ± SD)	Denture Use
Benign (no dysplasia)	9	11	47.56 ± 8.33	46.5 ± 8.36	46.45 ± 8.16	^1^ FPD (*n* = 6)
Mild/moderate dysplasia	11	9	59.18 ± 7.01	58.7 ± 6.9	58.7 ± 6.90	FPD (*n* = 3), ^2^ RPD (*n* = 2)
Carcinoma in-situ	14	6	70.1 ± 5.34	68.9 ± 4.8	70.1 ± 5.34	FPD (*n* = 3), RPD (*n* = 4), ^3^ CD (*n* = 2)
OSSC	12	8	69.1 ± 5.35	68.23 ± 4.98	69.05 ± 5.02	FPD (*n* = 2), RPD (*n* = 5), CD (*n* = 4)
Total	46	34	63.34 ± 11.19	58 ± 11.45	61.07 ± 11.54	*n* = 31 (38.7%)

^1^ FPD: Fixed partial dentures; ^2^ RPD: removable partial denture; ^3^ CD: Complete dentures.

**Table 5:** The distribution of *Candida* sp. according to gender and histopathological diagnosis.

Histopathological Diagnosis	*C. albicans*		*C. glabrata*		*C. kruseii*		*C. tropicalis*		*C. parapsilosis*	
	Males (*n*)	Females (*n*)	Males (*n*)	Females (*n*)	Males (*n*)	Females (*n*)	Males (*n*)	Females (*n*)	Males (*n*)	Females (*n*)
Benign (no dysplasia)	6	2	5	6	4	2	-	-	1	2
Mild/moderate dysplasia	11	7	6	5	4	5	6	1	1	2
Carcinoma in-situ	9	3	2	4	7	3	-	1	-	-
OSSC	4	2	3	5	5	3	2	-	-	1
Total	30 *	14	16	20 *	20	13	8	2	2	5

* Statistically significant difference.

**Table 6:** The number of samples with the presence of different *Candida* sp. in each histopathological group.

Numbers of Positive Samples						
Histopathological Diagnosis	Number of Samples	*C. albicans*(%)	*C. glabrata* (%)	*C. kruseii* (%)	*C. tropicalis* (%)	*C. parapsilosis* (%)
Benign	20	8 (40)	11 (55)	6 (30)	ND	3 (15)
Mild/moderate dysplasia	20	18 (90) *	11 (55)	9 (45)	7 (35) *	3 (15)
Carcinoma in-situ	20	12 (60)	6 (30)	10 (50)	1 (5)	ND
OSSC	20	6 (30)	8 (40)	8 (40)	2 (10)	1 (5)
Total	80	44 (55)	36 (45)	33 (41.25)	10 (12.5)	7 (8.75)

ND: Not detected, * Statistically significant difference.

## Data Availability

The data presented in this study are available on request from the corresponding author. The data are not publicly available due to legal restrictions and privacy.
